# Pathway potency in methylation altered network reveals lung cancer branching evolution and hallmarks

**DOI:** 10.1016/j.isci.2026.116730

**Published:** 2026-07-10

**Authors:** Zhilong Mi, Jiasen Zhang, Fuxun Li, Qingcai He, Taihang Huang, Mao Li, Zhiming Zheng, Binghui Guo

**Affiliations:** 1 Institute of Artificial Intelligence, Beihang University, Beijing 100191, China; 2 Beijing Advanced Innovation Center for Future Blockchain and Privacy Computing, Beihang University, Beijing 100191, China; 3 Key Laboratory of Mathematics, Informatics and Behavioral Semantics (LMIB), Beihang University, Beijing 100191, China; 4 State Key Laboratory of Complex & Critical Software Environment (SKLCCSE), Beihang University, Beijing 100191, China; 5 Beijing International Center for Mathematical Research, Peking University, Beijing 100871, China; 6 Chongqing Research Institute of Big Data, Peking University, Chongqing 400041, China; 7 School of Mathematical Sciences, Beihang University, Beijing 100191, China

**Keywords:** pathway potency, methylation altered networks, branching evolution, sample state classification, cancer hallmarks

## Abstract

The inability to profile the dynamic evolution and heterogeneity of cancer cells presents a significant barrier to effective early screening for lung cancer. By mapping pathway interactions within and beyond gene systems, one can decode their synergistic interactions underlying molecular stability and state transitions. To this end, we propose an efficient approach that derives pathway potency characterization from methylation altered sample specific networks. Applying it to lung adenocarcinoma samples uncovers a seven-state trajectory across three branching points. We show that while patient survival tends to decline within each state, it improves when the cancer transitions to a new state at branching points, which offers a more dynamic view of progression. Our approach also identifies the top feature pathways that distinguish different cancer states. Given the marked heterogeneity in pathway interplay patterns, we propose this method holds promise for decoding cancer complexity and eventually resolving critical issues in early screening.

## Highlights

• MASS-Path assesses pathway potency from methylation altered sample specific networks
• MASS-Path captures lung cancer’s branching evolution via tumor trajectory inference
• MASS-Path’s discrimination power is validated by top feature pathways between states
• Heterogeneity and plasticity in pathway interplay patterns inform cancer hallmarks

## Graphical abstract

**Figure:**
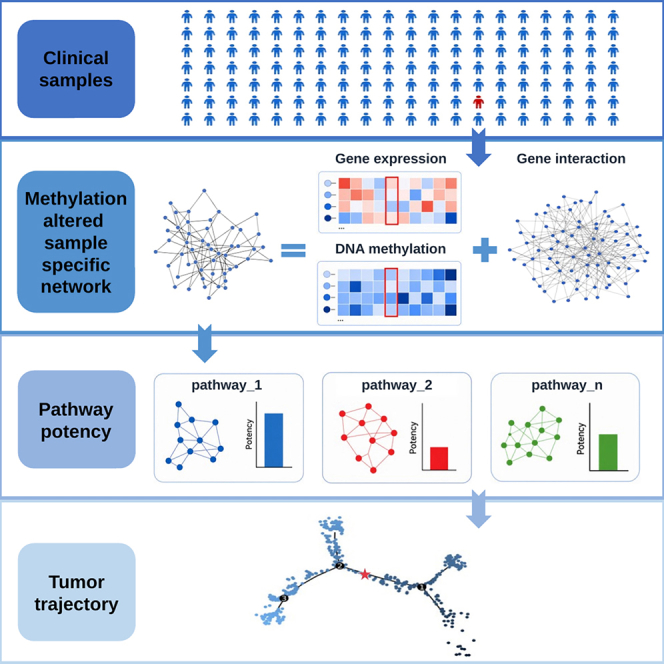

## Teaser

Bioinformatics; Systems biology; Cancer

## Introduction

Lung cancer remains the most common malignancy, claiming approximately 1.8 million lives annually, making it the leading cause of cancer-related mortality.^1^ The complexity of lung cancer stems from its genotype-phenotype heterogeneity. The AJCC cancer staging system is the primary system used by healthcare professionals to describe the extent and spread of a cancer. It undergoes regular updates to incorporate the latest scientific and clinical findings. However, it’s crucial to understand a core oncological principle that the stage assigned at the initial diagnosis of cancer is fixed and does not change, representing a snapshot of the cancer’s extent at the time of diagnosis based on the TNM system (tumor, nodes, metastasis). The developmental relationship between cancer stages exhibits significant variations across different editions of the AJCC cancer staging system. Both the lag in data and the updating of standards point to the issue of cancer phenotype heterogeneity. Diverse driver mutations and intricate staging systems collectively challenge treatment optimization and prognostic accuracy.^2^ In response, initiatives like the Human Genome Project II (HGP2) promote cross-omics integration for precision medicine through in-depth insights into gene-cell cross-scale evolution.^3^ Decoding the behavioral complexity of molecules embedded in data is critical to addressing the challenges of misdiagnosis and overmedication in early screening.

The effective inference of gene network structures based on observable data helps reveal the causal mechanisms of early-stage cancer development and the spatiotemporal dynamic patterns of cancer progression. Direct causal inference metrics such as partial mutual information and partial correlation are proposed for network topology inference, sample specific network construction, and personalized biomarker characterization.^4^^,^^5^ Integrating multi-view prior information into gene expression network reconstruction enables effective identification of subtypes of glioblastoma, breast cancer, and other cancers.^6^^,^^7^ However, focusing on causal mechanisms between static variables overlooks the fact that cancer cell populations evolve a cross-scale mechanism of branching differentiation. The biological pathway informed approaches suggest that cancer progression is involved in dynamic crosstalk relationships between pathways.^8^

Pathway activity inference is dedicated to exploring cancer phenotype heterogeneity and plasticity.^9^^,^^10^ Identification of significant changes in the activity of specific pathways has revealed molecular stability dynamics.^11^ Algorithms such as Pathifier infers pathway disruption scores for each tumor sample by measuring the deviation of each pathway’s behavior from normal.^12^ SEPA introduces the information-theoretic entropy in generating pathway representation for each patient and is empirically demonstrated on several datasets.^13^ Moreover, changes in the balance of interactions between specific pathways illuminate the transformation and reversal of cancer states.^14^ Accordingly, iPath is devised for identifying critical perturbed pathways as prognostic biomarkers, even if the perturbation occurs only in a subset of tumor samples.^15^ To advance our knowledge of cancer manifestations, it is necessary to investigate synergistic interactions within and beyond gene systems.^16^

Recent studies have highlighted the importance of collecting multi-omics and epigenetic profiling.^17^^,^^18^^,^^19^ Integrating DNA methylation into network modeling provides insights into cancer development, opening new avenues for therapeutic strategies.^20^^,^^21^ Temporal discordance between epigenetic remodeling and transcriptional turnover creates regulatory latency in current multi-omics pipelines.^22^ Current network models remain constrained by linear cascade assumptions, overlooking the combinatorial effects of methylation on transcriptional bursting, translational efficiency, and protein modification dynamics.^23^ Hence, a weighted gene network reconstruction approach has been proposed to explore the dynamic interplay between gene expression and epigenetic regulation.^24^ Still, organ specific variability and interindividual variability in DNA methylation profiles remain major challenges in characterizing consistent prognostic biomarkers.^25^

**Figure 1:**
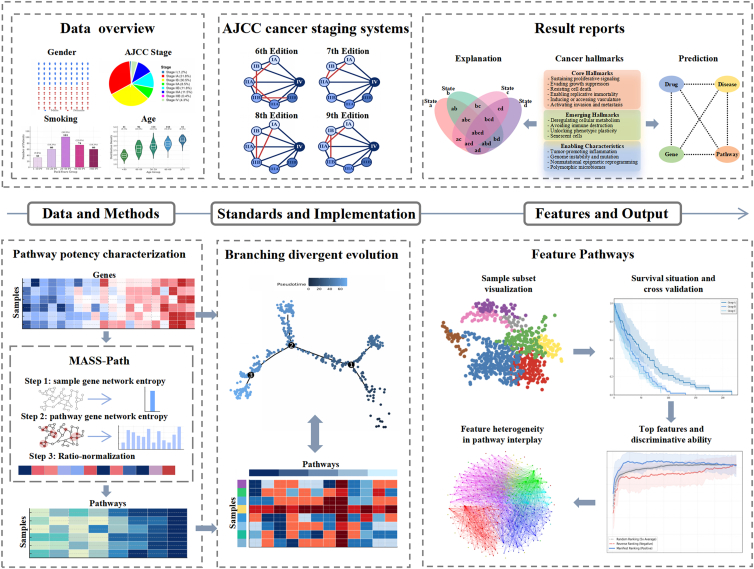
Overview of this work The staging systems for cancer patients originate from clinical characteristics, whereas disease progression trajectories identified through updates are diverse and not constant. From methylation altered sample specific networks, we derive pathway potency characterization and discover an entropy-increase evolution of branching differentiation in lung cancer samples. For the new branch states, we separately obtain the top feature pathways that characterize the sample subsets and demonstrate strong discriminative ability.

In this work, we propose an efficient approach named MASS-Path, which derives pathway potency characterization from methylation altered sample specific networks, as illustrated in Figure 1. In the TNM classification system, a single-grade elevation of only component (T, N, or M) can cause a given tumor stage to diverge into multiple distinct stages. However, it remains too abstract to reveal what significant functional or qualitative shifts such changes reflect in tumor biology. A one-to-one mapping between the internal architecture of gene interactions and the global behavior of the gene network cannot be assumed. Using a coarse-graining strategy that preserves functionality, our framework initially assigns to each pathway and each sample a potency measure via Markov chain entropy (MCE)^26^ in the context of human gene network, respectively. To assess directional order changes in entropy of a local pathway function, the ratio of the pathway gene network entropy to the sample gene network entropy is calculated. Since biological systems are highly ordered, the establishment and maintenance of which is a process with relatively low local entropy. A ratio greater than 1 indicates an increase in entropy, while a ratio less than 1 suggests a decrease in entropy. Therefore, we discover entropy increase evolution of branching differentiation in lung cancer samples. For the new branch states, we separately obtain the top pathway features that characterize the sample subsets and demonstrate strong discriminative ability. In addition, the heterogeneity in distinct pathway interplay patterns links cancer hallmark principles to functional outcomes, providing insights into cross-scale genotype-phenotype heterogeneity.

## Results

### Branching divergent evolution from spatiotemporal coupling of gene expression and pathway potency

**Figure 2:**
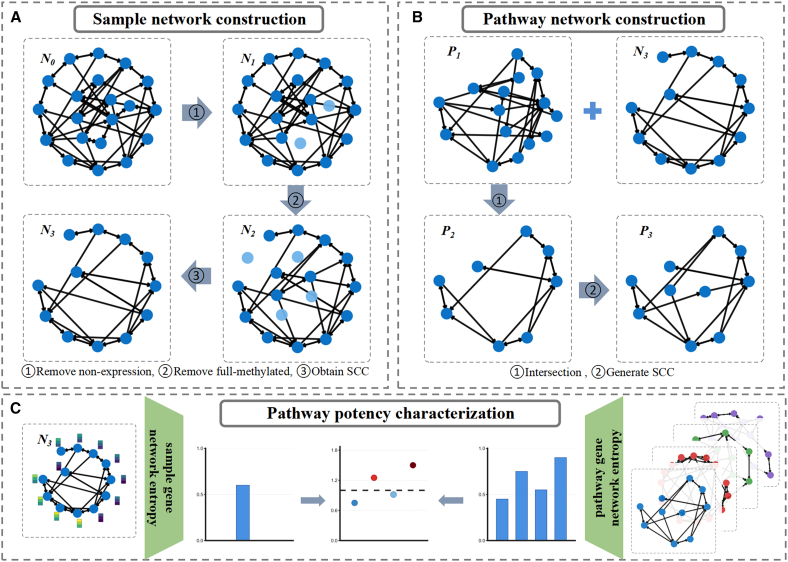
Methylation altered sample specific networks and pathway potency characterization (A) For each sample, the specific network N3 is constructed from the reference network N0, through removing non-expression, removing full-methylated, and obtaining the largest strongly connected component (SCC). (B) Generate an SCC as pathway gene network P3 centered on the intersection of N3 and pathway genes. (C) The ratio of the pathway gene network entropy to the sample gene network entropy serves as the pathway potency characterization. The relationship between the value and 1 reflects either an increase or decrease in entropy of a local pathway function.

**Figure 3:**
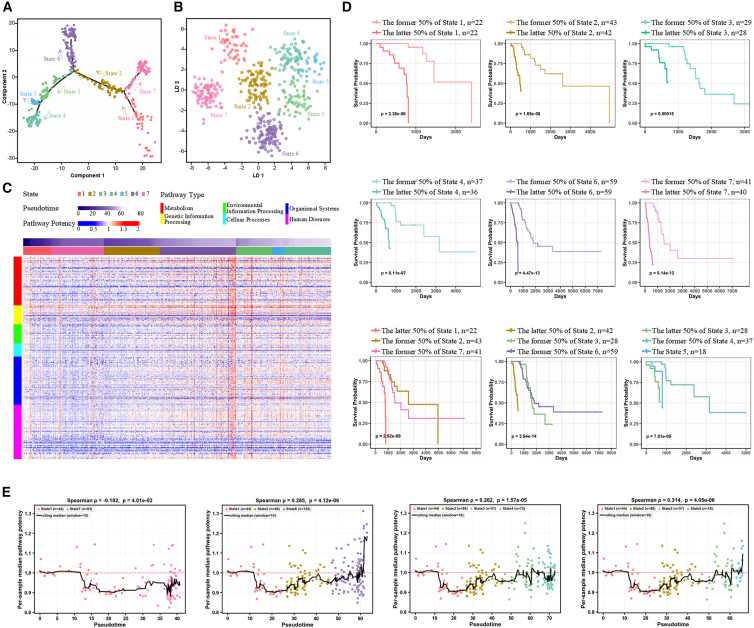
Branching divergent evolution of LUAD (A) Pseudotime analysis orders samples along a trajectory according to their gene expression profiles, resulting in their classification into 7 states encompassing 3 branching points. Arrows indicate the direction of pseudo-time progression. (B) The spatial distribution of samples via linear discriminant analysis based on pathway potency, exhibiting corresponding alignment patterns with pseudotime evolution in (A), as indicated by matching colors. (C) The heatmap of pathway potency of samples. The columns represent individual samples, grouped by States identified through pseudotime analysis and ordered by increasing pseudotime within each State. The top annotation bars indicate each sample’s pseudotime value and state label. The rows represent KEGG pathways. The left-side color annotation bar indicates the KEGG pathway category. (D) Kaplan-Meier survival curves for samples within different states and between states at branching points. (E) Median pathway potency versus pseudotime for each trajectory, and spearman correlation coefficients are provided. P-values were calculated using the log-rank test.

Here, we introduce the role of genes and pathways, derive a vector characterization of pathway potency in the samples as illustrated in Figure 2, more details are available in STAR Methods. It provides evidence of accompanying entropy increase of local pathway functions which aligns with the cancer branching divergent evolution observed at the gene level (shown in Figures 3A–3C). By sorting heterogeneous samples along a pseudo-time axis based on transcriptional state similarity, LUAD samples are classified into seven states encompassing three branching points, as illustrated in Figure 3A. In addition to demonstrating pseudo temporal evolution, we also verify that the spatial distribution of samples via linear discriminant analysis based on pathway potency, exhibiting corresponding alignment patterns, as illustrated in Figure 3B. Given the pathway potency of samples, one can readily observe which functional pathways within the sample exhibit increased or decreased entropy values. It is clearly illustrated through the heatmap in Figure 3C, the disease states are first arranged in ascending order of their average pseudotime values. Within each state, samples are ordered by increasing pseudotime. Red indicates that pathway potency exceeds 1, signifying an increase in local entropy, while blue denotes the opposite. For samples on each trajectory in Figure 3A, we plot the median pathway potency against sample pseudotime and calculate the Spearman correlation coefficient as illustrated in Figure 3E. The level of abstraction we adopt captures emergent biological phenomena like oscillations and switch-like behaviors. Besides, the proportions of samples with a median pathway potency exceeding 1 for each state are as follows: state 1 (25.0%), state 7 (16.9%), state 2 (18.2%), state 6 (38.3%), state 3 (31.6%), state 5 (55.6%), and state 4 (30.7%). A landmark discovery is that samples from the tail of state 6 exhibits pathway efficacy values exceeding 1 in almost all cases. Specifically, this concerns 12 samples, of which 8 are recorded as dead, all presenting with N2 metastasis or T2 stage. The remaining 4 samples are recorded as alive, though it remains unclear whether they have migrated to a higher stage, thereby masking the disease progression outcome.

### The divergent survival outcome associated with state transitions

Kaplan-Meier survival curves for the seven states of samples are shown in Figure 3D. Specifically, samples within each state are divided into former 50% and latter 50% groups, with the exception of state 5 due to insufficient sample size. A consistent observation is that survival outcomes for the former 50% samples are markedly superior to those of the latter 50% samples, with the log rank test showing significance. Furthermore, survival curves for samples from adjacent states are plotted at three bifurcation points, revealing marked disparities. Notably, survival outcomes progressively deteriorate within each state; however, upon transitioning to a new state after the bifurcation point, survival outcomes actually improve. This reflects temporal heterogeneity within states, indicating that samples within the same state still exhibit evolutionary differences. As pseudo-time progresses, pathway entropy values gradually accumulate, functional disorder intensifies, and survival risks escalate. Meanwhile, although samples in new states following branching originate from prior states, they develop distinct biological characteristics due to pathway reprogramming through methylation regulation. This appears as enhanced potency in certain pathways and diminished potency in others, ultimately leading to marked divergence in survival curves.

### Characterizing top feature pathways in sample state classification

**Figure 4:**
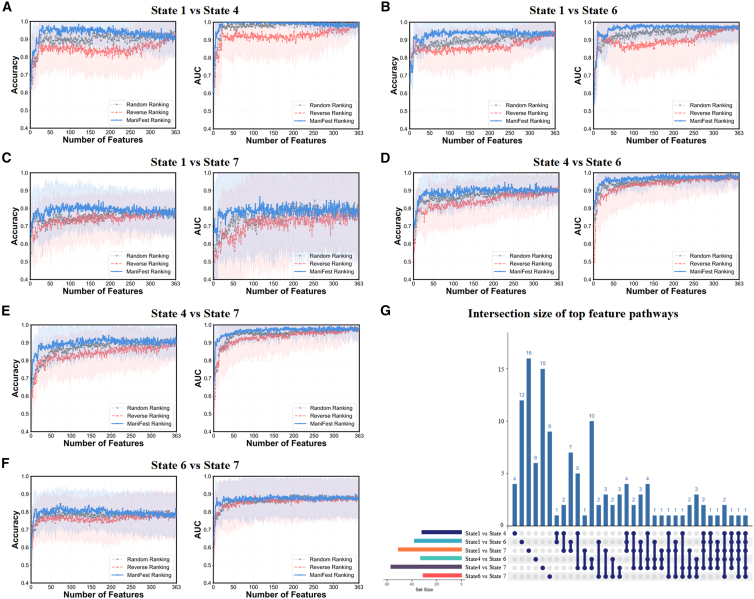
Top feature pathways and discriminative ability in sample state classification (A–F) For the two given state samples, pathway features are ranked using a supervised feature selection algorithm ManiFeSt, with performance evaluated by accuracy and AUC values. Where state 1 is the starting state, and state 4, 6, 7 are the terminal states. The blue curve represents the pathway arranged in ManiFeSt order, the red curve represents the pathway arranged in reverse order, and the gray curve represents the pathway arranged in random order. The top feature pathways are obtained when accuracy reaches its peak. Data are represented as mean ± SEM. (G) Visualization of the top feature pathway sets and intersections. The detailed top feature pathway sets and intersections are available in Table S3.

Samples are classified into seven states based on pathway potency characterization, where state 1 is the starting state, state 2 and 3 are transitional states, state 4, 6, and 7 are terminal states. State 5 contains too few samples to be considered a specialized state. A crucial question then arises: which pathways constitute the core characteristics that distinguish these states? The initial and terminal states are the focus of attention. Therefore, for each pair of state samples, all 363 pathways are scored and ranked using a supervised feature selection algorithm ManiFeSt. By sequentially incorporating pathways from the sorted list into the classification model and evaluating its performance through accuracy and AUC values, the top features are identified, as illustrated in Figures 4A–4F.

A total of 130 pathways are identified as top features for different tasks. Notably, pairwise classification performance between state 1, 4, and 6 is excellent. Among these, 32 pathways serve as top features for state 1 and 4, achieving an accuracy of 96.7% and an AUC value of 100%. 38 pathways serve as top features for state 1 and 6, achieving an accuracy of 96.7% and an AUC value of 100%. 33 pathways serve as top features for state 4 and 6, achieving an accuracy of 90.5% and an AUC value of 95.2%. And the distinction from state 7 is relatively good. Specifically, 51 pathways serve as top features for state 1 and 7, achieving an accuracy of 82.1% and an AUC value of 78%. 57 pathways serve as top features for state 4 and 7, achieving an accuracy of 91% and an AUC value of 96.3%. 31 pathways serve as top features for state 6 and 7, achieving an accuracy of 81.8% and an AUC value of 86.4%.

### The intersection of top feature pathways enhances heterogeneity

The intersection of various top feature pathway sets is illustrated in Figure 4G. Due to the differing functional roles of pathways, some are features of multiple tasks. Pathways like Other types of O-glycan biosynthesis pathway (hsa00514), Glycosphingolipid biosynthesis pathway (hsa00601), Tuberculosis pathway (hsa05152) belong to 5 out of 6 sets. D-Amino acid metabolism pathway (hsa00470), Biosynthesis of cofactors pathway (hsa01240), Mucin type O-glycan biosynthesis pathway (hsa00512), Pyrimidine metabolism pathway (hsa00240), GnRH signaling pathway pathway (hsa04912), Linoleic acid metabolism pathway (hsa00591) belong to 4 out of 6 sets. These are primarily metabolic pathways, whose involvement in substance metabolism is intrinsically linked to disease processes.

Many other pathways are only specific to a single task. To distinguish states 1 and 4, Arachidonic acid metabolism pathway (hsa00590), Neutrophil extracellular trap formation pathway (hsa04613) are identified as top features. And it has been found that sustained lung inflammation and the accompanying formation of NETs could convert dormant cancer cells to aggressive lung metastases.^27^ Besides, arachidonic acid plays a crucial role in the immune evasion, and therapeutic resistance through its diverse metabolites.^28^^,^^29^ To distinguish atates 1 and 6, NOD-like receptor signaling pathway (hsa04621), T cell receptor signaling pathway (hsa04660) are identified as top features, and it has been reported that they engage in a critical cross-talk that determines the overall anti-tumor immune response within the tumor microenvironment of lung cancer.^30^^,^^31^ To distinguish states 1 and 7, Calcium signaling pathway (hsa04020), PD-L1 expression and PD-1 checkpoint pathway in cancer (hsa05235) are identified as top features, and it is found that calcium is a key upstream regulator of PD-L1 expression and release, and combining of both serves integrated prognostic value.^32^ To distinguish States 4 and 6, PI3K-Akt signaling pathway (hsa04151), Th1 and Th2 cell differentiation (hsa04658) are identified as top features. Because the PI3K-Akt pathway shapes an microenvironment that inhibits Th1 cell-mediated anti-tumor immune responses and potentially shifts the Th1/Th2 balance toward a Th2-dominant pro-tumor state, thereby aiding tumor immune evasion. To distinguish states 4 and 7, Osteoclast differentiation pathway (hsa04380), Parathyroid hormone synthesis, secretion and action pathway (hsa04928) are identified as top features, reminding the distant metastasis and systemic complications of lung cancer, particularly bone metastases and hypercalcemia.^33^ Besides, choline metabolism in cancer (hsa05231) supports rapid synthesis of biological membranes by tumor cells to promote migration. Dilated cardiomyopathy pathway (hsa05414) highlights the molecular mechanisms of cardiac injury associated with potential cardiac side effects arising from treatments for lung cancer.^34^ To distinguish states 6 and 7, MAPK signaling pathway (hsa04010), JAK-STAT signaling pathway (hsa04630), Natural killer cell mediated cytotoxicity pathway (hsa04650) are identified as top features. Since the MAPK pathway suppresses immune function, indirectly diminishing the activity of natural killer cells and T cells. Conversely, when natural killer cells successfully recognize and attack tumor cells, the JAK-STAT pathway enhances anti-tumor immune responses.

### Distinct pathway interplay patterns highlight plasticity

**Figure 5:**
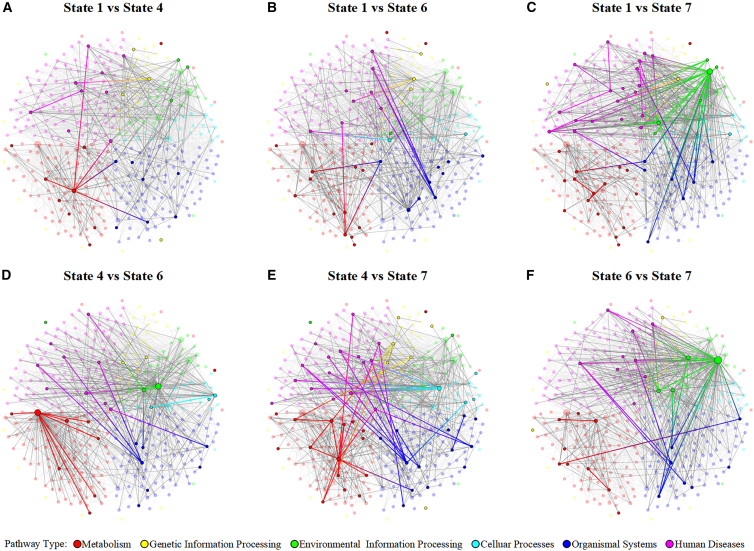
Top feature pathways in the pathway-related network (A-F) Interplay among top-ranked feature pathways identified from Figure 4. The pathway-related network is constructed based on relationships documented within KEGG PATHWAY. Each node in the network represents a biological pathway, with directed edges capturing the regulatory relationships between pathways. Full colored nodes denote the top feature pathways, with colors indicating their pathway types. The remaining pathways are labeled in shades. Edges connecting two top feature pathways are highlighted in color, while all other relationships are presented in gray.

The fundamental point is that pathways do not operate in isolation but rather form a highly interconnected and complex signal network that facilitates continuous information exchange between pathways. Here, we construct a pathway-related network based on relationships documented with KEGG PATHWAY. As illustrated in Figure 5, each node in the network represents a biological pathway instead of an individual gene, with directed edges capturing the regulatory relationships between pathways as specified by the KEGG database. Full colored nodes denote the top feature pathways that distinguish different sample states, corresponding to the results in Figure 4, with colors indicating their pathway types. The remaining pathways are labeled in shades. To emphasize key interactions, edges connecting two top feature pathways are highlighted in color, while all other relationships are presented in gray.

Figure 5A shows that top pathways distinguishing state 1 and 4 are not hubs except Citrate cycle (TCA cycle) pathway (hsa00020). It has evolved from a simple energy generator into a complex metabolic control center. Related pathways, such as Glucagon signaling pathway (hsa04922) and D-Amino acid metabolism pathway (hsa00470) act as inputs and regulators for this hub. Proximal tubule bicarbonate reclamation (hsa04964) manages the waste output, while Diabetic cardiomyopathy pathway (hsa05415) and Cushing syndrome pathway (hsa04934) contribute to complications.

Figure 5B shows the association between the top pathways distinguishing state 1 and 6 as follows: Oxidative phosphorylation pathway (hsa00190) and Hormone signaling pathway (hsa04081) provide fundamental energy for growth. NOD-like receptor signaling pathway (hsa04621) and Th17 cell differentiation pathway (hsa04659) shape a pro-tumor inflammation and immunosuppressive microenvironment. The T cell receptor signaling pathway (hsa04660) determines the efficacy of adaptive anti-tumor immunity. Focal adhesion pathway (hsa04510) executes the final lethal step of invasion and metastasis.

Figure 5C shows the primary characteristic of the top pathways distinguishing state 1 and 7 lies in the environmental information processing pathways, such as Calcium signaling pathway (hsa04020), Notch signaling pathway (hsa04330), Hippo signaling pathway (hsa04390), cGMP-PKG signaling pathway (hsa04022), TGF-beta signaling pathway (hsa04350). These pathways are closely related to cancer and interconnect a wide array of organ system pathways and human disease pathways.

Figure 5D shows the main difference of state 4 and 6 is the metabolism, as deduced from the major Metabolic pathways (hsa01100) and its numerous associated components. Besides, there are the aforementioned PI3K-Akt signaling pathway (hsa04151), cAMP signaling pathway (hsa04024), Antigen processing and presentation pathway (hsa04612). In addition, Renin-angiotensin system pathway (hsa04614) and GnRH signaling pathway (hsa04912) act as dual regulators. The Th1 and Th2 cell differentiation pathway (hsa04658) and Efferocytosis pathway (hsa04148) define the immunological landscape. Autophagy pathway (hsa04140) dictates the cellular adaptation response.

Figure 5E demonstrates the intricate interplay between various pathways distinguishing State 4 and 7. Inactivation of p53 signaling pathway (hsa04115) potentially downregulating Antigen processing and presentation pathway (hsa04612) to aid tumor cells in achieving invisibility. Th17 cell differentiation pathway (hsa04659) and IL-17 signaling pathway (hsa04657) drive chronic inflammation, which may be affected by Intestinal immune network for IgA production pathway (hsa04672) through immune regulation.

Figure 5F demonstrates that the main focus of the top pathways distinguishing state 1 from state 7 centers on the MAPK signaling pathway (hsa04010), JAK-STAT signaling pathway (hsa04630), TGF-beta signaling pathway (hsa04530) being the core drivers and regulators of tumor development. Besides, cAMP signaling pathway (hsa04024) and GnRH signaling pathway (hsa04912) act as potential counter-signals that could be harnessed therapeutically. While the Natural killer cell mediated cytotoxicity pathway (hsa04650) and Antigen processing and presentation pathway (hsa04612) represent the primary anti-tumor defenses, which are actively suppressed in the tumor microenvironment.

### Top feature pathways contribute to hallmarks of lung cancer

The Hallmarks of cancer represent the functional capabilities acquired by all cancer cells during their development, whereas pathways constitute the molecular mechanisms through which these capabilities are realized. In Table 1, we assign the 130 top feature pathways into the 14 hallmarks, which belong to three broad categories: core hallmarks, emerging hallmarks, and enabling characteristics.^35^ A single hallmark is often enabled by multiple pathways. Conversely, a single dysregulated pathway can contribute to multiple hallmarks. These pathways possess redundancy through intricate interconnected networks. Inhibiting one pathway can lead to compensatory activation of another, and targeted therapies are designed to specifically inhibit these pathways.

**Table 1:** The top feature pathways incorporate into hallmarks of cancer

Category	Hallmark	Pathways (KEGG IDs)
Core hallmarks	sustaining proliferative signaling	hsa04010 hsa04020 hsa04022 hsa04024 hsa04081 hsa04151 hsa04630 hsa04912 hsa04914 hsa04928 hsa04934 hsa05017 hsa05200 hsa05215 hsa05217 hsa05219 hsa05221 hsa05224 hsa05225 hsa05226 hsa05231
	evading growth suppressors	hsa04115 hsa04350
	resisting cell death	hsa04010 hsa04140 hsa04148 hsa04151 hsa04215 hsa04390
	enabling replicative immortality	hsa03018 hsa03030 hsa03050
	inducing or accessing vasculature	hsa04270 hsa04330 hsa04614 hsa05412 hsa05414 hsa05415 hsa05205
	activating invasion and metastasis	hsa04380 hsa04390 hsa04510 hsa04611 hsa04814 hsa05205
Emerging hallmarks	deregulating cellular metabolism	hsa00020 hsa00061 hsa00062 hsa00071 hsa00190 hsa00220 hsa00240 hsa00260 hsa00280 hsa00350 hsa00380 hsa00410 hsa00450 hsa00470 hsa00511 hsa00512 hsa00513 hsa00514 hsa00515 hsa00531 hsa00532 hsa00533 hsa00541 hsa00590 hsa00591 hsa00601 hsa00603 hsa00630 hsa00650 hsa00730 hsa00770 hsa00830 hsa00910 hsa00970 hsa01100 hsa01230 hsa01240 hsa01250 hsa01523 hsa02010 hsa04122 hsa04922 hsa04923 hsa04964 hsa04975 hsa04978 hsa04980 hsa05231
	avoiding immune destruction	hsa04612 hsa04650 hsa04658 hsa04659 hsa04660 hsa04672 hsa05235 hsa05330 hsa05332
	unlocking phenotypic plasticity	hsa04330 hsa04713 hsa04720 hsa04916
	senescent cells	hsa04710
Enabling characteristics	tumor-promoting inflammation	hsa04145 hsa04613 hsa04621 hsa04623 hsa04657 hsa05120 hsa05321
	genome instability and mutation	hsa03430 hsa03450 hsa03460
	nonmutational epigenetic reprogramming	hsa03082 hsa05202 hsa05203
	polymorphic microbiomes	hsa03264 hsa05132 hsa05133 hsa05142 hsa05143 hsa05144 hsa05146 hsa05150 hsa05152 hsa05161 hsa05164 hsa05165 hsa05167 hsa05168 hsa05170 hsa05171

**Figure 6:**
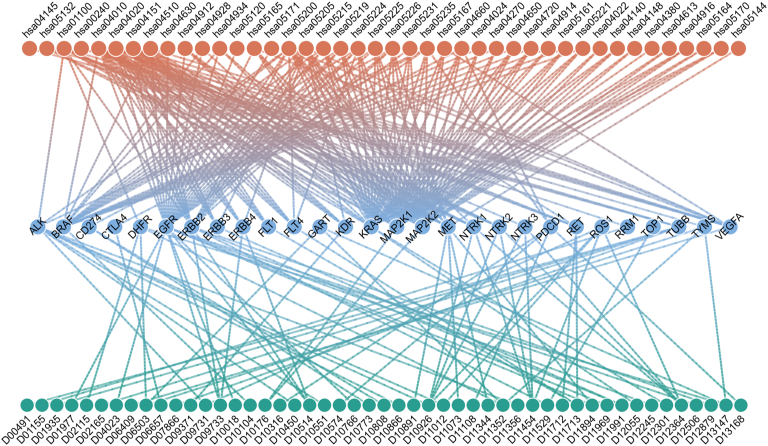
Targeted interactions between drugs, genes, and top feature pathways for lung cancer The KEGG database catalogs 49 drugs targeting lung cancer, which act upon 28 genes (families) and 42 top feature pathways.

As illustrated in Figure 6, totally 49 drugs are documented in KEGG for lung cancer, targeting 28 genes (families) and 42 pathways. A number of pivotal genes are involved in multiple pathways and simultaneously serve as targets for various therapeutic drugs. It is essential to perform molecular profiling to identify driver mutations in genes, such as EGFR, KRAS, ALK, ROS1, MET, RET, ERBB2(HER2), BRAF, NTRK1, and MAP2K1 (MEK1), as targeted therapies for these genes can directly counteract the hallmark of sustaining proliferative signaling. Drugs targeting genes involved in angiogenesis, such as VEGFA, KDR (VEGFR2), and FLT1 (VEGFR1) can directly oppose the hallmark of inducing or accessing vasculature. Immune checkpoint inhibitors targeting PDCD1(PD-1), CD274(PD-L1), and CTLA4 can directly counteract the hallmark of avoiding immune destruction. Agents targeting DHFR primarily address the hallmark of resisting cell death, while those targeting TUBB interfere with the hallmark of enabling replicative immortality. Additionally, inhibitors of GART act against the hallmark of deregulating cellular metabolism by disrupting nucleotide synthesis and metabolic adaptation in cancer cells.

## Discussion

The development of MASS-Path addresses a key limitation of static frameworks in capturing the branching divergent evolution of tumors. By constructing sample specific networks informed by methylation alterations, our approach advances beyond population-average profiling to provide a multi-scale functional view of individual tumors. Integrating DNA methylation, a dynamic and plastic regulator of gene expression, into pathway-level analysis enables deeper understanding of sample state mechanisms. This approach facilitates quantitative assessment of pathway potency within each sample, offering a dynamic perspective for observing tumor heterogeneity. When applied to LUAD, MASS-Path delineates a tumor progression trajectory comprising seven distinct states across three branching points, shedding new light on the emergence of phenotype heterogeneity and plasticity, and revealing how epigenetic reprogramming drives tumor diversification.

A particularly intriguing observation is the divergent survival outcome associated with state transitions. While patient survival progressively deteriorates within a stable state, it improves upon transition at a bifurcation point. This finding may indicate that the transitional process itself represents a vulnerable phase or critical reset point for the tumor system. It challenges the linear paradigm of relentless progression and implies that strategies aimed at forcing or trapping tumors at specific bifurcation points may alter adverse prognoses more effectively than therapeutic interventions targeting static states.

Considering conventional pathway analysis methods such as ssGSEA, which primarily quantify static enrichment based on gene expression abundance, we applied ssGSEA to the LUAD dataset to obtain pathway-level rankings of gene importance across samples. For each of the 363 KEGG pathways, we performed non-parametric statistical comparisons between sample groups representing distinct states. Specifically, for each pair of states, we extracted ssGSEA scores for each pathway and applied the two-sided Mann-Whitney U test to evaluate significant differences in score distributions between the two groups. To control for false positives due to multiple comparisons, we adjusted the resulting *p* values using the Benjamini-Hochberg procedure for each condition pair. To evaluate the consistency and complementarity of the top feature pathways obtained based on MASS-Path and ssGSEA, we summarized their intersections in Table S1. Both MASS-Path and ssGSEA leverage sample specific information for feature selection. MASS-Path selects a smaller number of features, the majority of which are validated by ssGSEA, while ssGSEA shows considerably greater feature redundancy. This observation indicates that through the integration of gene expression, methylation, and interaction information, MASS-Path achieves a reduction in redundancy while retaining essential biological signals, resulting in a more refined feature selection and a more concise, interpretable feature set suitable for downstream applications.

In The Cancer Genome Atlas (TCGA), samples with the normal type 11A are more accurately termed adjacent normal tissues rather than truly healthy controls. Molecular evidence indicates that these samples are often not entirely normal, largely attributable to the field effect, resulting in altered gene expression, low-level mutations, and epigenetic dysregulation. The 11A normal samples and their matched 01A tumor samples share a common origin but diverge in fate, reflecting distinct evolutionary trajectories arising from the same genetic background. Pseudotime captures transcriptomic dysregulation and cell state shifts, both of which evolve along the disease trajectory and drive tumor progression. Notably, a previous study has identified a position along the pseudotime where tumor purity drop sharply, a trend invisible when samples are ordered by the time from primary tumor occurrence.^36^ This pattern is consistent with the characteristics of 11A normal samples which have high values of pseudotime. In our study, all 11A samples of are clustered at the tail of state 6 and are associated with poor prognosis, suggesting originate from patients with severe disease progression, likely reflecting cases where adjacent normal tissues are collected due to poor clinical response.

The high dimensionality of omics data, with its attendant risk of spurious correlations, presents a major obstacle to deciphering true biological signals and disease evolutionary heterogeneity. Consequently, reconstructing branching trajectories capable of capturing non-linear, state-dependent dynamics is pivotal. This facilitates the identification of critical regulatory interactions emerging at transition thresholds, the comprehension of which is central to elucidating varied clinical outcomes and resistance mechanisms, thereby promoting effective disease progression modeling and risk stratification. Our method stratifies patients into distinct dynamic states with differing progression risks and identifying the pathway features that drive each state. This yields a mechanism-aware model that links dynamic network configurations to clinical outcomes, providing a basis for stage-specific intervention strategies.

The limited feature dimensions of the currently available clinical data precluded an in-depth correlation analysis with specific histological subtypes in this study. The pathway-informed characterization approach essentially embeds unknown dimensions as latent variables by explicitly incorporating structural interaction information. In addition, the validity of this approach is further substantiated by the alignment of the top feature pathways with the hallmarks of cancer. We propose that immune cell infiltration is linked to the branching of tumor states, as evidenced by the prominence of immune-related pathways among our high-frequency features. These pathways cover key aspects of anti-tumor immunity. Immune recognition is mediated by the Antigen processing and presentation pathway (hsa04612). Effector cell differentiation involves the Th1/Th2 (hsa04658) and Th17 (hsa04659) differentiation pathways. Functional execution is carried out through the T cell receptor signaling pathway (hsa04660), the IL-17 signaling pathway (hsa04657), and the Natural killer cell mediated cytotoxicity pathway (hsa04650). By modulating these pathways, tumor cell states directly influence the extent and effectiveness of immune infiltration, laying a theoretical groundwork for pathway-based tumor state classification and immunotherapy response prediction.

The discriminative power of MASS-Path is further demonstrated by its ability to identify top feature pathways distinguishing different states. We have observed a clear correspondence between alignment patterns derived from gene expression-based pseudotime analysis and pathway potency-based linear discriminant analysis with the superior clustering clarity highlighting the cross-scale insights gained from integrating pathway-level information. To further validate this robustness, we applied a supervised feature selection method combined with a Random Forest classifier to score and rank all 363 pathways for each pair of state samples. The classification model exhibits exceptional performance in distinguishing the primary evolutionary states, achieving excellent pairwise separation among states 1, 4, and 6, and relatively good distinction from state 7. This underscores its practical utility in locating key mechanistic drivers and potential biomarkers.

To validate our findings, we performed an independent analysis on the TCGA-LUSC dataset adhering to the standard MASS-Path pipeline. As the two major subtypes of non-small cell lung cancer, LUAD typically originates from peripheral mucus-secreting cells and is enriched with EGFR mutations, while LUSC arises from squamous metaplasia of central bronchial epithelium, characterized by TP53 and PI3K pathway alterations. As illustrated in Figure S1, pseudo-time is assigned to each sample, and LUSC samples are classified into five states and two branching points. For the new branch states, we obtain the top pathway features that characterize their corresponding sample subsets and exhibit strong discriminative ability. The pathway potency signatures derived from MASS-PATH reveals that LUAD follows multiple parallel trajectories characterized by dynamic multicellular niche transitions. In contrast, LUSC exhibits a dominant linear trajectory marked by defined cell-state transitions from precancerous to cancerous stages. These findings underscore the capability of this approach in resolving subtype-specific evolutionary trajectories.

Given the marked heterogeneity in pathway interplay patterns revealed in this work, we anticipate MASS-Path will be become a pivotal tool for enhancing the diagnostic accuracy of early-stage tumor, thereby addressing issues of misdiagnosis and overmedication. Moving from descriptive to causal-mechanistic understanding hinges on advances in dynamical causal inference. Explicitly modeling the perturbation-response mechanisms underlying empirical state transitions remains a focus of emerging perturbation-omics. Achieving reliable counterfactual predictions that simulate changes in a patient’s disease trajectory under specific interventions will profoundly transform the direction of treatment design and personalized medicine. Future work will focus on validating this model in larger prospective cohorts and exploring its applicability to other cancer types, with the ultimate goal of translating these insights into personalized and effective treatment strategies.

### Limitations of the study

This study constructs a methylation altered sample specific network for each sample using paired RNA-seq and DNA methylation data. While this successfully captures entropy-based pathway potency dynamics, its assumption of a pseudotemporal progression inferred from transcriptional similarity may not fully reflect the complex features of tumor evolution. Integration of multi-omics data such as copy-number variations and additional epigenetic markers would be valuable to further investigate how pathway potency manifests across diverse molecular contexts. Moreover, the reconstruction of tumor progression trajectories is limited by sample size. In particular, detailed characterization of state 5 is not feasible in this work, which may result in an underestimation of rare evolutionary trajectories. These underscore the necessity for future validation in larger, multi-center datasets and the integration with additional omics layers to enhance the model’s robustness and clinical applicability.

## Resource availability

### Lead contact

Requests for further information and resources should be directed to and will be fulfilled by the lead contact, Zhilong Mi (mizhilong9@buaa.edu.cn).

### Materials availability

This study did not generate new unique reagents.

### Data and code availability

• The human gene interaction data are public available and can be download at BioGRID,^37^ specifically version 4.4.203. The RNA-seq data, DNA methylation data are public available and can be download from the TCGA project via the GDC data portal,^38^ specifically from the LUAD and LUSC projects. The human pathways and targeted interactions between drugs, genes, and pathways are public available and can be download at KEGG,^39^ specifically from the Release 115.0, July 1, 2025. Processed data needed to evaluate the conclusions of the current study are present in the manuscript and the supplemental information, which are also uploaded to Zenodo (https://doi.org/10.5281/zenodo.19116531). All other data reported in the manuscript will be shared by the lead contact upon request.
• The codes are uploaded to GitHub (https://github.com/Zhangjs02/MASS-path). We provide a README file serving as a comprehensive and practical entry point for reproduction. It documents the code organization structure, required input parameters, expected output results, graph-level reproduction workflow, and supplemental information.
• Any additional information required to reanalyze the data reported in this paper is available from the lead contact upon request.

## Acknowledgments

This work was supported by the Brain Science and Brain-like Intelligence Technology-10.13039/501100018537National Science and Technology Major Project (grant no. 2021ZD0201302), the 10.13039/501100001809National Natural Science Foundation of China (grant no. 62388101), the Fundamental Research Funds for the Central Universities, and Beijing Advanced Innovation Center for Future Blockchain and Privacy Computing.

## Author contributions

Conceptualization, Z.M. and B.G.; methodology, Z.M., J.Z., and F.L.; investigation, J.Z. and F.L.; validation, Z.M., Q.H., M.L., and T.H.; writing – original draft, Z.M., J.Z., and F.L.; writing – review and editing, Z.M., Q.H., Z.Z., and B.G.; funding acquisition, Z.M., Z.Z., and B.G.; supervision, Z.M. and B.G.

## Declaration of interests

The authors have declared that no competing interests exist.

## STAR★Methods

### Key resources table

**Table:** 

REAGENT or RESOURCE	SOURCE	IDENTIFIER
**Deposited data**		

Human gene interaction network	BioGRID	https://downloads.thebiogrid.org The human gene interaction data are freely available and obtained from BioGRID,^37^ specifically version 4.4.203.
TCGA project multi-omics data	TCGA via GDC Data Portal	https://portal.gdc.cancer.gov/ The RNA-seq data and DNA methylation data used in this study are obtained from The Cancer Genome Atlas (TCGA) database,^38^ specifically from the LUAD and LUSC projects.
Human pathways	KEGG	https://www.genome.jp/kegg/pathway.html The human pathways and targeted interactions between drugs, genes and pathways are public available and can be download at KEGG,^39^ specifically from the Release 115.0, July 1, 2025.
The intersection of top feature pathways obtained based on MASS-Path and ssGSEA	This paper	Table S1
sample specific gene networks and within pathway networks	This paper	Table S2
Pathway potency values by sample and pathway	This paper	Table S3
The top feature pathway sets and intersections	This paper	Table S4
Data Repository	This paper	https://doi.org/10.5281/zenodo.19116531

**Software and algorithms**		

R version 4.5.0	CRAN	https://cran.r-project.org/src/base/R-4/R-4.5.0.tar.gz
Python version 3.10	Python Software Foundation	https://www.python.org
Conda environment	Anaconda	environment.yml
Monocle2	Bioconductor	https://bioconductor.org/packages/monocle/
DDRTree	CRAN	https://cran.r-project.org/package=DDRTree
Random Forest	scikit-learn	https://scikit-learn.org/
Code Repository	This paper	https://github.com/Zhangjs02/MASS-path

### Experimental model and study participant details

This study did not involve experimental models.

### Method details

#### Overview of MASS-Path

The AJCC cancer staging system is subject to regular updates to reflect the latest clinical evidence, leading to substantial variations across editions, such as the 6th (2002), 7th (2009), 8th (2016), and 9th (2020). Figure 1 illustrates these variations in the stepwise evolution of cancer states, where blue edges represent common routes and red edges denote version-specific routes. The interplay between lagged real-world data and these evolving standards reveals the fundamental issue of of heterogeneity and plasticity in cancer phenotypes. Given that the initial stage assignment is a fixed snapshot at diagnosis, it fails to reflect this heterogeneity over time. We therefore propose to characterize pathway potency as a means to infer the trajectory of tumor progression, aiming to unlock deeper insights from existing data.

In As shown in Figure 1, the staging systems for cancer patients originate from clinical characteristics, whereas disease progression trajectories identified through updates are diverse and not constant. From methylation altered sample specific networks, we derive pathway potency characterization and analyze tumor progression trajectories in lung cancer and evaluated its applicability. By integrating RNA sequencing with DNA methylation profiling, we perform the following analyses: sample/pathway strongly connected network construction and MCE computation, batch pathway MCE calculation, pseudotime inference, top feature pathway selection and validation, pathway interplay pattern visualization. Building upon the hallmarks of cancer as a guiding framework, we explore their relationships with top feature pathways to enable a more precise characterization of distinct disease states. By analyzing the intersections of these pathway sets across different contexts, we can identify mechanisms of heterogeneity and plasticity that are conserved across cancer states. These insights not only deepen our understanding of how hallmark capabilities are acquired and maintained, but also provide a basis for predicting associations among genes, pathways, diseases, and drugs, thereby supporting the discovery of potential therapeutic targets and biomarkers.

#### Directed human gene interaction network

In this work, directed human gene interaction is collected from BioGRID (version 4.4.203),^37^ where the bait-prey direction serves as the foundation for constructing the human gene network. This network comprises 20195 human genes and 942241 directed interactions. Particularly, the largest strongly connected component, denoted as *N*_*0*_, possesses 16092 nodes and 902432 directed edges, and forms the reference network for all downstream network instantiations.

#### TCGA multi-omics data preprocessing

We chose the typical LUAD and LUSC data from TCGA,^38^ which includes 485 and 378 samples, respectively. In addition, multi-omics data of both RNA-seq (60660 genes) and DNA methylation (27578 genes) are obtained. In detail, the gene level expression data are normalized by the upper-quartile FPKM. The probe-level methylation data following standard Illumina gene calling are aggregated into gene-level beta values by averaging probes for the same gene. It has revealed that gene methylation in most samples exhibits a bimodal distribution pattern, with peaks occurring at 0.1 and 0.9, respectively.^26^

#### KEGG pathway collection

Pathways from KEGG are collected, representing our knowledge of molecular reactions under both normal and perturbed states.^39^ We select 363 human pathways, which are divided into six major types as follows: metabolism, genetic information processing, environmental information processing, cellular processes, organismal systems, and human diseases. In total, 9439 human genes are covered, with each gene participating in an average of 4.17 pathways.

#### Methylation altered sample specific network

As illustrated in Figure 2(A), for each sample, the specific network is constructed as follows. From the reference network *N*_*0*_, genes with missing values or zero expression are excluded to yield the largest connected network *N*_*1*_. Subsequently, fully methylated genes (where *β* ≥ 0.6) are filtered to obtain the methylation altered network *N*_*2*_, under the assumption that full methylation induces transient gene suppression. Ultimately, the largest strongly connected component of *N*_*2*_ is designated as *N*_*3*_, from which the Markov Chain Entropy is subsequently derived. For each sample, the number of nodes and edges in corresponding network *N*_*3*_ is available in Table S1.

Concurrently, the pathway gene network is constructed as presented in Figure 2(B). Let the intersection of *N*_*3*_ and genes for a specific pathway *P*_*1*_ be denoted as *P*_*2*_. We note that the majority of gene relationships within pathways are not identified in the interaction data, indicating these relationships represent indirect associations between pathway genes. Consequently, an algorithm Algorithm 1 is devised within the context of *N*_*3*_ to generate a strongly connected component *P*_*3*_ centered on *P*_*3*_, thereby characterizing the pathway network. For each pathway in each sample, the number of nodes and edges in corresponding network *P*_*3*_ is also available in Table S1.

Algorithm 1 **Strongly connected pathway network generation**

**Input:** Directed graph *G*=(*V*,*E*) representing pathway network

**Output:** Set of strongly connected components *SCCs*

1 Step 1: First DFS on *G*

 Initialize empty stack *S*

 **foreach** vertex *v*∈*V* **do**

2 **if** *v* not visited **then**

3 DFS (*G*,*v*,*S*)// Push *v* to *S* after recursion finishes

4 Step 2: Transpose Graph

 Construct *G*^*T*^ by reversing all edges in *G*

5 Step 3: Second DFS on *G*^*T*^

 Initialize *SCCs*=*ϕ*

 Mark all vertices as unvisited

 **while** *S not empty* **do**

6 *v*←Pop(*S*)

 **if** not visited **then**

7 *C*←DFS(*G*^*T*^,*v*)

 Add *C* to *SCCs*

8 **Return** *SCCs*

The algorithm can be briefly summarized by the following formulas:

(Equation 1) $$\begin{array}{c} T\left(v\right)=post\left(v;DFS\left(G\right)\right),S=\left\langle v_{1},\ldots ,v_{n}\right\rangle \;T\left(v_{1}\right)\geq \ldots \geq T\left(v_{n}\right)\text{.} \end{array}$$

Perform the first Depth-First Search (DFS) on the original graph G=(V,E) to obtain the completion time τ(v) for each vertex, and form a sequence/stack S of vertices in descending order based on this.

(Equation 2) $$\begin{array}{c} C_{k}=ReachG\left(v_{k}\right)\cap U_{k-1},U_{k}=U_{k-1}\setminus C_{k}\text{,} \end{array}$$

(Equation 3) $$\begin{array}{c} SCCs={\left\{C_{k}\right\}}_{k=1}^{m}\text{.} \end{array}$$

Perform a second DFS on the transposed graph *G*^*T*^ in the order of *S*. Let *U*_*0*_ = *V*. Each time, take the currently unallocated vertex *v*_*k*_ as the source, intersect its reachable set with *U*_*k-1*_ to obtain a strongly connected component *C*_*k*_, and remove it from the unallocated set; eventually, all SCCs are obtained.

#### Markov Chain Entropy

To quantify the level of behavioral complexity inherent in the data of each sample, the Markov Chain Entropy (MCE) is calculated, which is a latent quantification model derived from the steady-state information flow within the network structure.^24^ The core hypothesis of MCE posits that the normalized gene expression profile (*π =* (*π*_*1*_*, π*_*2*_*, …, π*_*nv*_)) of a single sample (where $\sum_{i=1}^{n_{V}}\;\pi_{i}=1$) represents the steady-state expression distribution of the gene network, and MCE is mathematically defined as the information entropy measuring global signal flow heterogeneity:

$$MCE=\max_{p_{ij}\geq 0}-\sum_{\left(i,j\right)\in \bar{E}}\pi_{i}p_{ij}\;\log\left(\pi_{i}p_{ij}\right)$$

(Equation 4) $$\begin{array}{c} subject\;to\;\sum_{j\in \bar{N}\left(i\right)}p_{ij}=1,i=1,\ldots ,n \end{array}$$

$$\sum_{j\in \bar{N}\left(i\right)}\pi_{i}p_{ij}=1,i=1,\ldots ,n$$

where *p*_*ij*_ denotes the transition probability from node *i* to node *j*, representing the propagation of signaling in the network, *π*_*i*_ denotes the stationary distribution probability of node *i*, determined by its gene expression level, *N*(*i*) represents the neighbor set of node *i*, including itself, $\bar{E}$ denotes the edge set, including both actual edges and self-loops (*i,i*).

To eliminate network size effects, MCE is normalized to the unit interval by the maximum possible entropy:

(Equation 5) $$\begin{array}{c} {MCE}_{norm}=\frac{MCE}{\log\left(d\right)},d=\sum_{i}\deg\left(i\right)\text{,} \end{array}$$

where deg(*i*) denotes the degree of node *i* (including self-loops), and *d* represents the total number of connections in the network.

#### Pathway potency characterization

For each sample *S*, its sample potency is calculated by MCE_*norm*_ on the sample gene network *N*_*3*_, and every single pathway potency is calculated by MCE_*norm*_ on the pathway gene network *P*_*3*_. Then, the ratio of the pathway gene network entropy to the sample gene network entropy is employed as an indicator to reflect directional order changes in entropy of a local pathway function. The formula is expressed as follows:

(Equation 6) $$\begin{array}{c} Potency\left(pathway\right)=\frac{{MCE}_{norm}\left(P_{3};\pi \right)}{{MCE}_{norm}\left(N_{3};\pi \right)} \end{array}$$

We characterize pathway potency within the sample *S* by (Potency(1), Potency(2), …, Potency(363)). Pathway potency quantifies the extent to which the activity pattern of a pathway is predictable versus random. A potency value of less than 1 indicates that the pathway exhibits regular activity, with low uncertainty regarding its subsequent state. In contrast, a potency value greater than 1 reflects a more diverse activity pattern, which is indicative of a dysregulated state. The detailed pathway potency in each sample is available in Table S2.

#### Branching divergent evolution

Pseudotime analysis is employed to uncover potential temporal progression and differentiation trajectories among samples. It is originally employed in single-cell studies, and may also be applied when a sufficient number of bulk samples are available.^40^ We adopted Monocle2 to order samples along a trajectory based on their gene expression profiles.^41^ In this analysis, states are defined as continuous segments along the inferred trajectory, each representing a distinct phase or branch of the biological process. To achieve this partitioning, the DDRTree algorithm first reduces high-dimensional gene expression data and learns a principal tree-like graph with branches. Samples are then projected onto this graph to determine their positions, and key topological features such as branch points are identified. Based on these branch points, the continuous graph is automatically partitioned into discrete segments, with each segment assigned a unique numeric identifier as its state. All samples residing in the same segment share that state value, indicating they are in a similar transitional stage or on the same developmental path. The input gene set consisted of 7844 genes from the methylation altered network *N*_*2*_. The resulting trajectory is segmented into distinct states, representing potential periods in cancer progression.

#### Survival analysis

To assess the clinical heterogeneity and plasticity of pseudotime inferred states, Kaplan-Meier survival analysis and Cox proportional hazards regression are conducted, enabling comparison of survival distributions across groups and estimation of hazard ratios, respectively.^42^ Each sample is assigned to a pseudotime inferred state, and survival curves are generated using the clinical follow-up data. The Log-rank test is applied to evaluate the statistical significance of survival differences across states. Within each state, samples are partitioned into two groups according to the median pseudotime value. Samples with pseudotime below the median are designated as former 50% samples, while those above the median are designated as latter 50% samples.

#### Top feature pathways and discriminative ability in sample state classification

To assess pathway importance across sample states, the supervised feature selection method ManiFeSt was employed to capture non-linear relationships between pathways and assign importance scores to each pathway.^43^ Random Forest is adopted as the classifier due to its robustness in handling high-dimensional data and its ability to model complex feature interactions. An ordered list of pathways are incrementally introduced into the classification model, increasing the number of selected features one by one from the top of the ranking until all pathways are included. To ensure robust evaluation, we employ repeated random subsampling validation, where 5% of the samples are selected as the test set and the remaining as the training set in each iteration. This process is repeated for 20 iterations, and the average performance across all iterations is reported as the final result. The classification performance are evaluated using accuracy and the area under the ROC curve (AUC).

(Equation 7) $$\begin{array}{c} ACC=\frac{TP+TN}{TP+TN+FP+FN} \end{array}$$

The horizontal axes of the ROC curve represent False Positive Rate (FPR):

(Equation 8) $$\begin{array}{c} FPR=\frac{FP}{FP+TN} \end{array}$$

The vertical axes of the ROC curve represent True Positive Rate (TPR):

(Equation 9) $$\begin{array}{c} TPR=\frac{TP}{TP+FN} \end{array}$$

The final expression is:

(Equation 10) $$\begin{array}{c} AUC\approx \sum_{k=1}^{m}\left(FPR_{k}-FPR_{k-1}\right)\cdot \frac{TPR_{k}+TPR_{k-1}}{2} \end{array}$$

#### Mann-Whitney U test

The Mann-Whitney U test is a non-parametric statistical method used to assess whether two independent samples originate from the same distribution. Unlike parametric alternatives such as Welch’s t-test, it does not assume normality of the underlying data, making it robust to outliers and suitable for small or unevenly sized samples. The test statistic is computed based on the ranks of all observations pooled from both groups.

Assuming there are two sets of data with sample sizes *n*_1_ and *n*_2_ respectively, mix all the observed values and sort them from smallest to largest, assigning ranks with the smallest being 1. Calculate the rank sums *R*_1_ and *R*_2_ for the two sets respectively. Calculate the *U* value:

(Equation 11) $$\begin{array}{c} U_{1}=n_{1}n_{2}+\frac{n_{1}\left(n_{1}+1\right)}{2}-R_{1},U_{2}=n_{1}n_{2}+\frac{n_{2}\left(n_{2}+1\right)}{2}-R_{2} \end{array}$$

#### Benjamini-Hochberg FDR

To address the multiple comparisons problem arising from simultaneously testing hundreds of pathways, the Benjamini-Hochberg procedure was applied to control the False Discovery Rate (FDR). The FDR represents the expected proportion of false positives among all results declared significant. Briefly, the raw *p*-values from all pathway-level tests within each state pair were ranked in ascending order, and adjusted *p*-values were computed as

(Equation 12) $$\begin{array}{c} \left(p_{\text{adj},\left(k\right)}=p_{\left(k\right)}\cdot m/k\right) \end{array}$$

where *m* is the total number of tests and *k* is the rank. A monotonicity constraint was enforced to ensure the adjusted *p*-values are non-decreasing. Pathways with adjusted *p*-values below 0.05 were considered statistically significant.

#### Top feature pathways and cancer hallmarks

To interpret the biological significance of the identified pathways, we link the top feature pathways to the cancer hallmarks framework. Cancer hallmarks are defined based on established literature, which categorizes them into 14 distinct capabilities across three broad categories: core hallmarks, emerging hallmarks, and enabling characteristics.^35^ For each pathway, its primary biological function is determined using KEGG pathway annotations and relevant supporting studies. Pathways are then assigned to one or more hallmarks according to the principle of functional consistency, that is, whether the dominant biological processes represented by the pathway directly contribute to the realization of a given hallmark capability. Given that many pathways participate in multiple biological processes, a one-to-many mapping between pathways and hallmarks is permitted. This mapping is performed through manual curation supported by literature evidence to ensure biological plausibility and reduce subjectivity. It serves as an interpretive layer to organize and biologically contextualize the top feature pathways presented in Table 1.

### Quantification and statistical analysis

Quantification and statistical analysis was performed by using Python and R. All results are presented as the mean or mean ± standard deviation (SD). Log-rank test and Mann-Whitney U test were employed. A *p-value* of less than 0.05 was considered statistically significant.

## Supplemental information

Document S1. Figure S1 and Table S1

Table S2. The number of nodes and edges in corresponding networks for each sample and within each pathway, related to Figure 2

Table S3. Pathway potency values by sample and pathway, related to Figure 3

Table S4. The top feature pathway sets and intersections, related to Figure 4
