# Supplementary material for: Pathway potency in methylation altered network reveals lung cancer branching evolution and hallmarks
Source: iScience. 2026 Jul 10;29(8):116730. doi: 10.1016/j.isci.2026.116730 (PMC13380764; doi:10.1016/j.isci.2026.116730)
Supplement: Document S1. Figure S1 and Table S1 [file mmc1.pdf]

iScience, Volume *29*

Supplemental information

Pathway potency in methylation altered network reveals lung cancer branching evolution and hallmarks

Zhilong Mi, Jiasen Zhang, Fuxun Li, Qingcai He, Taihang Huang, Mao Li, Zhiming Zheng, and Binghui Guo

Supplementary Figure S1, Tables S1

Supplementary Figure S1. Independent analysis on the TCGA-LUSC dataset adhering to the standard MASS-Path pipeline. The pathway potency signatures derived from MASS-PATH reveals that LUSC exhibits a dominant linear trajectory. LUSC samples are classified into five states and two branching points. For the new branch states, we obtain the top pathway features that characterize their corresponding sample subsets and exhibit strong discriminative ability.

Supplementary Table S1. The intersection of top feature pathways obtained based on MASS-Path and ssGSEA.

Supplementary Figure S1. Independent analysis on the TCGA-LUSC dataset adhering to the standard MASS-Path pipeline.

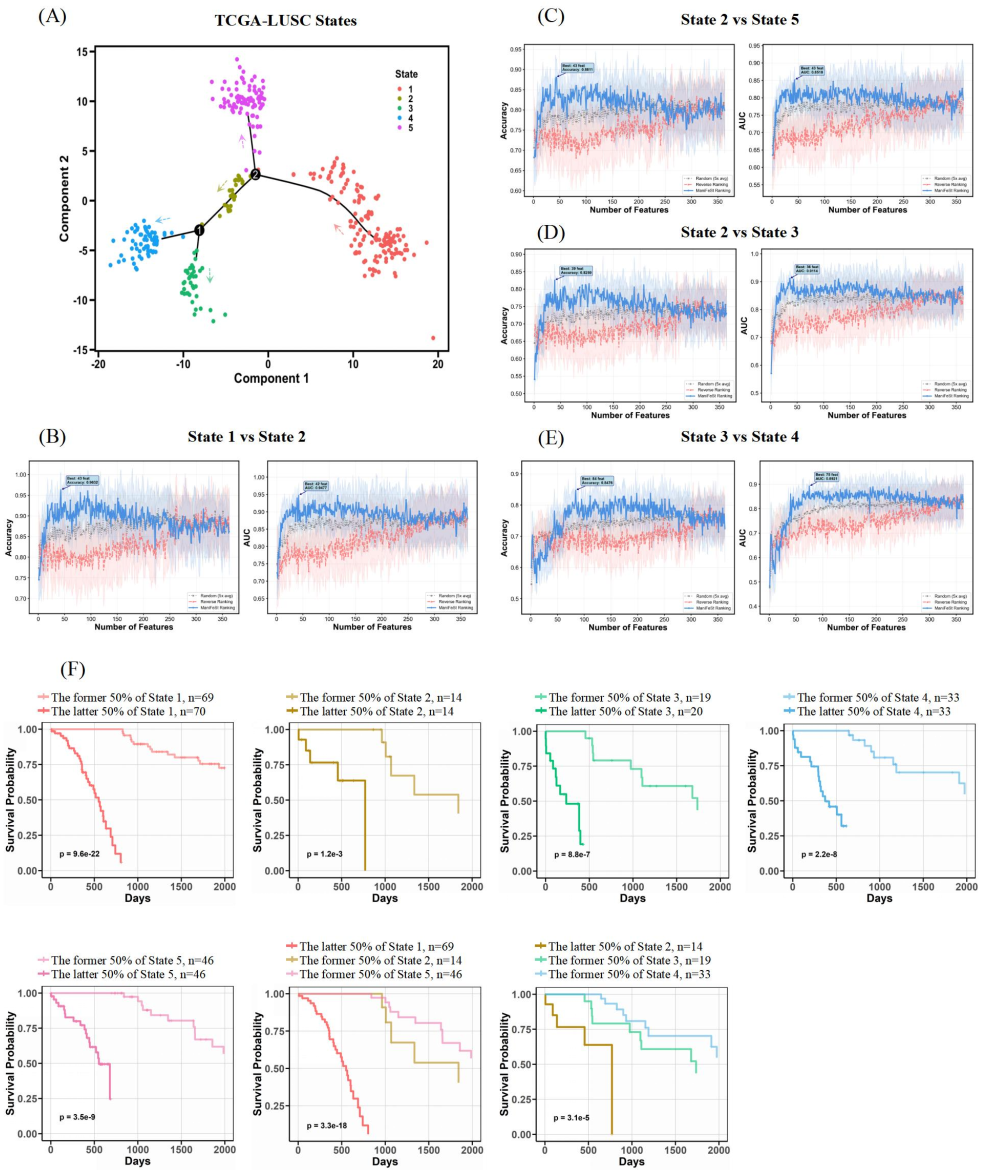

Supplementary Table S1. The intersection of top feature pathways obtained based on MASS-Path and ssGSEA.

**LUAD State**	**Top feature pathways**			
	**MASS-Path**	**ssGSEA**	**Intersection**	**Proportion**
State1 vs State4	32	75	16	50.00%
State1 vs State6	38	88	21	55.30%
State1 vs State7	32	86	16	50.00%
State4 vs State6	33	107	17	51.50%
State4 vs State7	30	124	18	60.00%
State6 vs State7	31	41	11	35.50%
